# A comparison of disease burden and symptoms with age among CoVid-19 patients from data in a Florida clinic

**DOI:** 10.6026/97320630017001

**Published:** 2021-01-31

**Authors:** Andrew P Collins, Chenan Andy Huang, Megan Ann Bernier, Naser Mubarak, Sami Hemaidan, Hadi Hemaidan, Ammar Hemaidan

**Affiliations:** 1 University of Central Florida College of Medicine Ringgold standard institution, 6850 Lake Nona Blvd Orlando, Orlando, Florida 32827 - 740, United States; 2 University of Central Florida College of Medicine Ringgold standard institution, Orlando, Florida, USA

**Keywords:** CoVid-19, symptoms, Florida clinic

## Abstract

Our knowledge of the disease burden and symptoms with age in COVID-19 patients is limited. Therefore, it is of interest to document the clinical aspect of this association with respect to the disease. We used the data of 3363 patients enrolled with an urgent care clinic in Volusia county, Florida for this study. Data shows difference in age among COVID-19 antibody (Ab) - positive patients (48.3 years, 95% CI = 46.9,49.7 years) and Ab-negative patients (46.1 years, 95% CI = 45.4, 46.8 years). However, disease burden by age is not significant on average. Nonetheless, COVID-19 positive patients between 40-69-years of age experienced the highest burden of disease and highest average number of symptoms. Thus, COVID-19 disease burden and number of symptoms experienced were highest among the 40-69-year-old patients. Those above the populations mean age of 46.4 years old were more likely to test positive for COVID-19.

## Background

The novel coronavirus disease 2019 (COVID-19) has evolved rapidly as a viral pandemic. Countries have been affected by the SARS (severe acute respiratory syndrome) CoV-2 virus since the end of 2019 [1]. During the original outbreak, high-density population centers with high mobility patterns, such as the New York metropolitan region, held as strongholds of COVID-19 with frequent viral transmission from person to person [2]. Now, as the disease spread in the United States has become less confined to dense population centers, we are seeing a more widespread distribution of the virus. Additionally, as stay-at-home measures and quarantine mandates are being lifted, the disease incidence is once again spiking; in Florida specifically, the June 2020 daily new cases spiked as high as 9585 in a single day [3]. The SARS-CoV-2 virus has shown unique behavior clinically and epidemiologically, as the disease has been reported to cause unusual symptoms in some patients and has also shown transmission from clinically asymptomatic patients who are unknowingly shedding the virus and exposing others to the risk of contracting COVID-19 [4-6]. Generally, populations that have not been infected are susceptible to viral strains they have not previously encountered. Furthermore, elderly patients with underlying diseases such as diabetes,hypertension, cardiovascular disease, cerebrovascular disease, and immune system disorders are at increased-risk of contracting the disease [7]. The elderly have been shown to be prone to severe illness associated with COVID-19, are admitted to intensive care units (ICUs) at a higher rate, and display a higher mortality rate than the disease in younger patients [8]. Therefore, it is of interest to document the clinical aspect of this association with respect to the disease.

## Methodology

### Study population:

A total of 3931 COVID-19 Ab tests were administered from April 1, 2020, through June 22, 2020. Of the total 3931 test results, we excluded 568, due to a lack of clinical information and/or absent values regarding patient demographics. Of the remaining 3363 subjects, we recorded 424 results positive for the presence of IgG and/or IgM against the SARS-CoV-2 virus. The tests were administered at the Advanced Medical Center urgent care clinic in Port Orange of Volusia county, Florida. The patients were stratified by age for analysis into the following ten-year-groups: 0-9 years, 10- 19 years, 20-29 years, 30-39 years, 40-49 years, 50-59 years, 60-69 years, 70-79 years, 80-89 years, and 90+ years. Data from the 80-89 and 90+ year-old groups were analyzed together due to the small sample size in each group.

### COVID-19 testing:

All positive COVID-19 tests were determined with laboratoryconfirmed testing methods.

COVID-19 testing was conducted using either of two tests: the CONFIRM BIOSCIENCES COVID-19 "Coronavirus" IgG/IgM Rapid Test Cassette or the RayBiotech Coronavirus (COVID-19) IgM/IgG Rapid Test Kit. Each of these tests utilizes samples of whole blood, serum, or plasma from the patients, which were taken at the test site on the day of testing. For the CONFIRM BIOSCIENCES test, when compared to the reference reagent, the positive agreement was 93.87% (95% confidence interval [CI] = 90.24, 96.46%), the negative agreement was 99.10% (95% CI = 97.70,99.75%), and the total agreement was 97.19% (95% CI = 95.65,98.26%). The kappa value of consistency analysis was 0.94 (95% CI = 95.65, 98.26). These results show that the test has a high degree of consistency and equivalent sensitivity and specificity in detecting COVID-19 [9]. The reported accuracy of the RayBiotech tests was 84.1% sensitivity and 92.3% specificity using the dual IgM and IgG test kit [10]. Information as to which testing method was utilized for each patient was not made available to us.

### Research methodology:

Upon arriving at Advanced Medical Center for antibody testing, patients received a survey to complete including questions about the symptoms they were experiencing. Data from the forms was grouped by patient age in ten-year intervals for analysis. The clinical symptoms evaluated on the forms for each age group were as follows: dry cough, fatigue, sputum production, shortness of breath, muscle or joint pain, sore throat, headache, chills, nausea, nasal congestion, diarrhea, hemoptysis, and conjunctival congestion. Since COVID-19 is classified as an influenza-like illness, patient disease burden was quantified by a scoring mechanism dependent on symptom presence and severity, similar to other trials assessing flu-like illness severity [11, 12]. Symptoms were classified in the severe grouping if they were a medical emergency or were likely to present in severe or atypical influenza-like illnesses (shortness of breath, hemoptysis, and diarrhea). Moderate severity symptoms were classified as general influenza-like illness symptoms (muscle/joint pain, chills, nausea, and conjunctival congestion). Mild severity symptoms were classified as typical upper respiratory infection clinical symptoms, similar to a common cold or mild influenza-like illness (dry-cough, fatigue, sputum production, sore throat, headache, and nasal congestion). High severity symptoms were assigned a value of five points, moderate severity symptoms were assigned three points, and low severity symptoms were given one point. This allowed for total symptom scores to be summed for each patient and age group in order to calculate and assess disease burden.

### Statistical analyses:

The program Python version 3.8.3 and SPSS Statistics 27 were used for all statistical analyses in this study. Disease burden, number of symptoms, known exposures, and positivity rate per age group were averaged and compared using a post-hoc analysis of variance (ANOVA) with 95% confidence intervals (CI), P value < 0.05, and a Bonferroni Correction to counteract multiple comparisons. A sample size calculation was utilized to obtain 80% power for a oneway analysis of variance F-tests (ANOVA) using an estimated effect size of 0.20, with 9 separate groups (stratified age groups), 5% significance level (α = 0.05), standard deviation (δ) = 0.10, and standard deviation of means (δμ) = 0.05. The calculation indicated a total of 72 participants, 8 per age group, to obtain a power of 82.85% to differentiate between age groups versus number of symptoms and COVID-19 disease burden. As such, our total sample size was 3363, and the smallest group (COVID-19 positive patients aged 0-9 and ≥ 80 years) consisted of 12 patients each.

## Results:

The age distribution in this data set was centered on the median age group of 40-49 years and the median age was 48 years (Table 1-see PDF). The mean age for the study population was 46.4 years (95% CI = 45.4, 46.8). There was a noted statistically significant difference in average age between the COVID-19 Ab-positive population (mean = 48.3 years, 95% CI = 46.9, 49.7) and the Ab-negative population (mean = 46.1 years, 95% CI = 45.4, 46.8). Our total data set and population of Ab-negative patients showed no statistically significant difference from the average age in Volusia county from 2018 (46 years). [13-14] The COVID-19 Ab-positive population did show a statistically significant difference between the means, indicating an older population is at increased risk of COVID-19 infection. The age data in our study population and each of the COVID-19 Ab-positive and negative groups demonstrated a negative skew. The largest group was the 50-59-year-old population, accounting for 18.9% of the total enrolled patients in the study.

Among the patients who tested positive for antibodies against COVID-19 (Table 2-see PDF), the 60-69-year-old population made up the greatest proportion (positivity count = 91, accounting for 21.5% of all positive tests). The 50-59-year-old group, which made up the largest proportion of enrolled patients in the study, only accounted for 70 positive tests (16.5% of total positivity). Both the 0-9 and ≥ 80-year-old patient groups accounted for only 12 positive tests (2.8% of total positivity) each. The ratio of positive test proportion to total test proportion demonstrates the highest positivity rate in the 60-69-year-old group. This indicates the highest likelihood for patients in this age group to test positive for the COVID-19 antibodies. The study population's highest positive rate (Figure 2) corresponded to the 60-69-year-old group (positivity = 17.6%), followed closely by the ≥ 80-year-old population (positivity = 16.4%), and the 0-9-yearold population (positivity = 16.2%). The 10-19-year-old population experienced the lowest Ab test positivity rate (10.3%). Statistically significant differences amongst age group to positivity rate were noted in the data. Known exposure rates per patient age group (Figure 3) were lowest among the 0-9-year-old population (44.6%), and highest among the 20-29, 30-39, and 50-59-year-old populations (61.0%, 61.0%, and 61.6%, respectively) (Table 2-see PDF). However, these high rates of known exposures did not correlate with increased COVID-19 Ab-positivity, as these three age groups each accounted for 3 of the 4 lowest Ab-positivity groups. Comparing the pooledage known exposure rates of COVID-19 Ab-positive patients (52.1%) to negative patients (57.8%) (Table 2-see PDF), there was no noted increase in known exposure in the Ab-positive population or statistically significant difference between groups. Of the 1443 patients who had noted no known exposure to COVID-19 infection, 14.07% tested Ab-positive. The 1920 patients that did report exposure to COVID-19 infection showed 11.51% Ab-positivity. The impetus for testing once suspecting COVID-19 exposure could be responsible for the decreased Ab-positivity rate amongst the group.

## Symptoms of COVID-19 Positive Patients:

**Figure 1:**
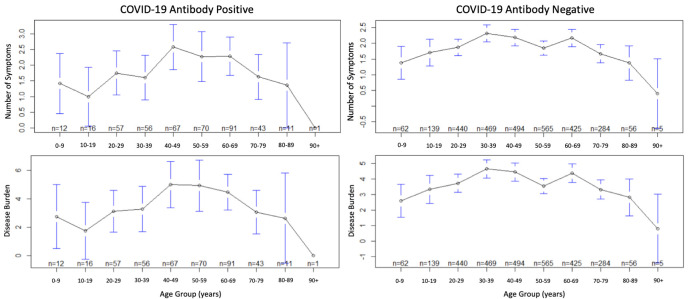
Age versus COVID-19 Antibody Positive and Negative Number of Symptoms and Disease Burden

**Figure 2:**
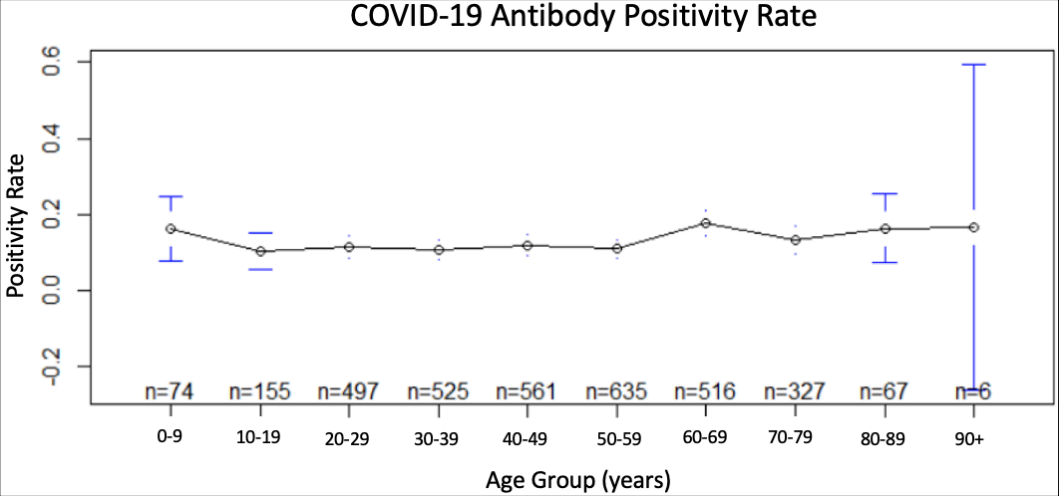
Age versus COVID-19 antibody positivity rate

**Figure 3:**
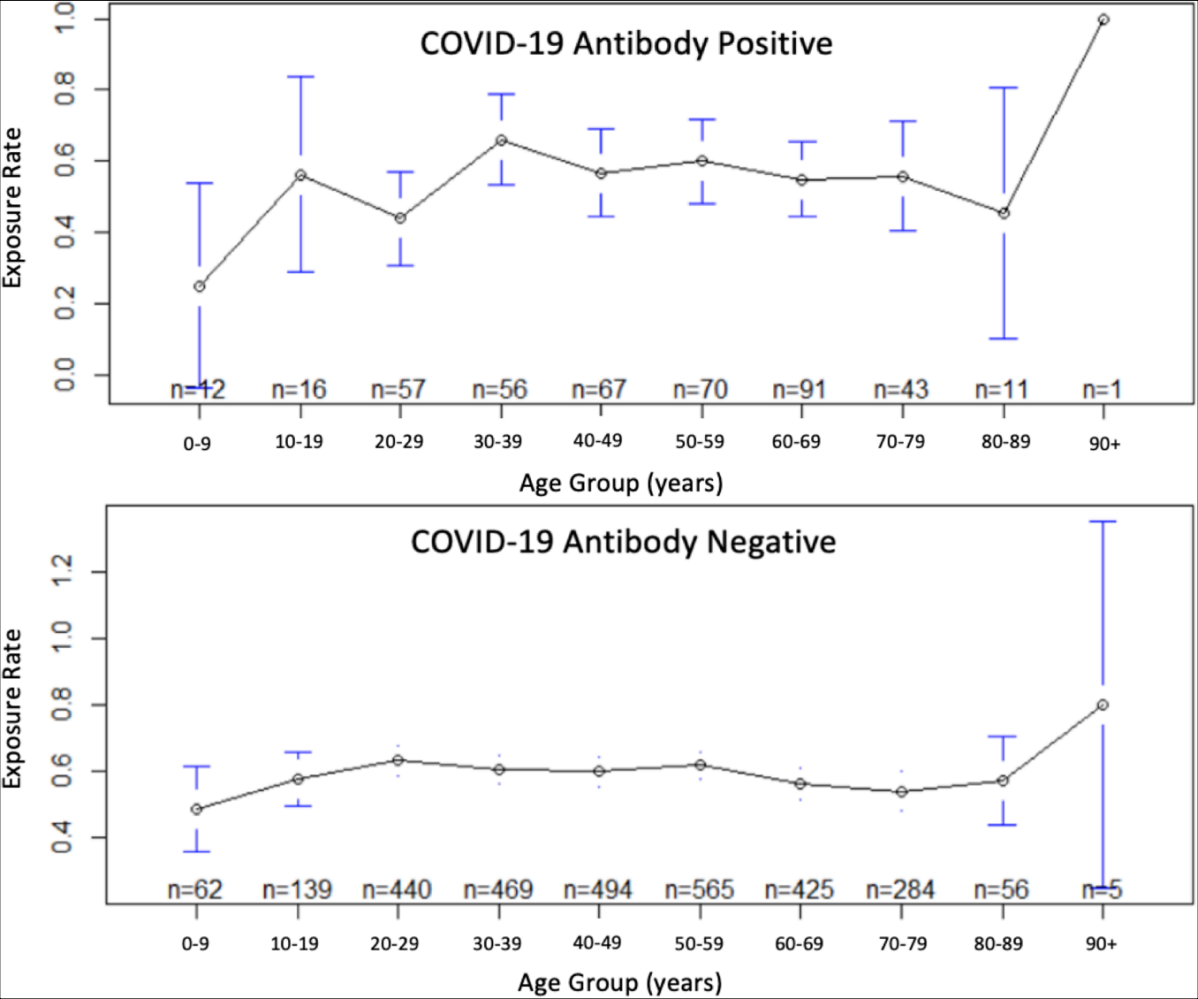
Age versus known exposure to COVID-19 for antibody positive and negative patients

The Ab-positive patients experienced an average of 2.00 symptoms per patient (Table 3 - see PDF). The population most likely to experience a greater number of symptoms was the 40-49-year-old group (2.58 symptoms). The groups least likely to experience high numbers of symptoms were the youngest and oldest. These results showed no statistically significant difference between age groups. The most common symptoms experienced by the COVID-19 Ab-positive patients varied amongst age groups. However, they were similar in that the most frequent symptoms were classified as mild severity for all populations (Table 2 - see PDF). Dry cough was the most common symptom in the 60-69, 70-79-year-old, and overall (pooled) populations, seen in 35.2%, 32.6%, and 23.9% of patients respectively. Fatigue was the most common symptom experienced by the 40-49 (37.3%), 50-59 (37.1%), and ≥ 80-year-old (33.3%) populations. Headache was the most commonly noted symptom in the 10-19 (25.0%), 20-29 (35.1%), and 30-39-year-old (30.4%) populations, while nasal congestion was the most common symptom in only the 0-9-year-old patient population but was experienced by 50% of these patients. Among the Ab-positive patients (Table 2 - see PDF), the most common severe symptom experienced was shortness of breath, seen in 12.7% of all patients. The next most common severe symptom was diarrhea, seen in 10.1% of patients, while the least likely was hemoptysis, appearing in only 0.89% of positive patients. The age group most likely to experience shortness of breath was the ≥ 80-year-old population, with a rate of 16.7%. Diarrhea was most commonly noted in the 50-59-year-old population, at 18.6%. Hemoptysis was only noted in three different age groups, 40-49, 50-59, and 60-69-years-old, with rates of 1.5%, 4.3%, and 2.2%, respectively. The most common moderate severity symptom was muscle/joint pain (Table 2 -see PDF), seen in 16.1% of COVID-19 Ab-positive patients. This symptom was noted most frequently in the 40-49-year-old population at a rate of 26.9%, while the least likely groups to experience this symptom were the 0-9 and ≥ 80-year-old population. Chills were the second most common moderate severity symptom, experienced by 11.8% of the Abpositive study group. Nausea and conjunctival congestion were each the least likely moderate severity symptom, experienced by 6.0% and 2.0% of the total Ab-positive population respectively. The COVID-19 Ab-positive group experienced mild severity symptoms more than any other symptom severity group (Table 2 - see PDF). Patients were commonly noted to be affected by dry cough (23.9%), fatigue (23.8%), headache (23.1%), and nasal congestion (21.4%). Amongst the various age groups, rates of dry cough reached as high as 35.2% (60-69-year-old population), fatigue in 37.3% (40-49-year-old population), headache in 35.1% (20-29-year-old population), and nasal congestion in 50.0% (0-9-year-old population). Sputum production and sore throat were less common mild severity symptoms. On average, both the Ab-positive and negative groups experienced a disease burden of 3.94 across all age groups (Figure 1). In the Ab-positive population, the highest disease burden impacted the 40-49 (burden of 5.00), 50-59 (burden of 4.93),and 60-69-year-old (burden of 4.46). Each of these groups experienced a respective increase in disease burden in the Abpositive population compared to the Ab-negative population. Of the Ab-positive population, only two groups experienced an average disease burden under 3.00, the 0-9 (burden of 2.75) and ≥ 80-year-old (burden of 2.42) populations. These groups also experienced the fewest number of symptoms on average, however the results showed no statistically significant difference between age groups. To assess symptom severity based on our burden of disease scoring mechanism, we took the quotient of the average disease burden and average number of symptoms present (Table 3 - see PDF). The result for the Ab-positive group was an average symptom severity of 1.97 versus 1.98 in the negative group. The 50-59 and 30-39-year-old populations of Ab-positive patients each experienced the highest average symptom severity (2.17 and 2.04, respectively). The 10-19 and 20-29-year-old Ab-populations experienced the lowest average symptom severity (1.75 and 1.78, respectively).

## Symptoms of COVID-19 Negative Patients:

On average, Ab-negative patients experienced 1.99 symptoms per patient (Table 3 -see PDF). Statistically significant differences between Abpositive and negative groups were noted. The two most common populations noted to experience >2 symptoms on average were the 40-49 (2.19 symptoms) and 60-69-year-old (2.17 symptoms) groups. Dry cough was noted as the most common symptom experienced in the 0-9 (27.4%) and 60-69-year-old (28.5%) groups (Table 2 - see PDF). Fatigue was the most common symptom in the 40-49 (30.6%), 50-59 (27.4%), 70-79 (24.3%), ≥ 80 (21.3%), and overall (pooled) (25.8%) populations. Headache was noted as the most common symptom experienced by the 10-19 (28.1%), 20-29 (25.9%), 30-39 (33.0%), and 40-49-year-old (30.6%) groups. Unique to the COVID-19 Abnegative group, a moderate severity symptom was the most common symptom present in the ≥ 80-year-old population. Of the 2939 COVID-19 Ab-negative patients, the most common severe symptom experienced was shortness of breath, noted in 13.4% of patients (compared to 12.7% of Ab-positive patients) (Table 2 - see PDF). The group that was most affected by shortness of breath was the 30-39 - year-old population, noting the symptom in 19% of patients. Diarrhea was the second most common severe symptom, seen in 9.3% of all Ab-negative patients (compared to 10.1% of patients in the Ab-positive group). Hemoptysis was only experienced by 0.44% of Ab-negative patients (compared to 0.89% of Ab-positive patients). The most common moderate severity symptom in Abnegative patients was muscle/joint pain, seen in 19.2% of this population (versus 16.1% in the Ab-positive patients) (Table 2 - see PDF). Like the Ab-positive patients, the next most common symptoms in the Ab-negative patients were chills (12.3% versus Ab-positive 11.8%), nausea (8.1% versus Ab-positive 6.0%), and conjunctival congestion (3.2% versus Ab-positive 2.0%). The Ab-negative group experienced the mild severity symptoms more than any other symptom severity classification (Table 2 - see PDF). The noted overall rates among all Ab-negative age groups were dry cough in 23.5% (versus 23.9% of Ab-positive patients), fatigue in 25.8% (versus 23.8% of Ab-positive patients), sputum production in 8.0% (versus 9.8% of Ab-positive patients), sore throat in 17.5% (versus 13.9% of Abpositive patients), headache in 22.9% (versus 23.1% of Ab-positive patients), and nasal congestion in 19.4% (versus 21.4% of Abpositive patients).

## Discussion:

The study population is likely a fair representation of the Volusia county demographics. The mean age of participants in the study was 46.4 years, and median 48 years, while the respective values for the county in 2018 were 46.0 and 47 years [13, 14]. Information on patient insurance carriers and access to care was not collected, and a socioeconomic status comparison to the county demographics was not obtained. COVID-19 Ab-positive patients were most likely to experience a greater number of symptoms between the ages of 40-69 years, and least likely in patients below 20 years and over 80 years. This trend was also noted in the average disease burden amongst positive patients, as the 40-69-year population demonstrated the highest burden of disease, while the 0-19 years and over 80-year-old population had the lowest burden. The strength of the human immune system has been noted to peak between the ages of 20-70 years, but before and after that range is weakened [15]. This likely explains the fewer symptoms and lower calculated disease burden as the immune response to the SARSCoV- 2 virus is not fully mounted in these young and elderly populations. In elderly patients over 70 years, they are likely to show a dysregulated adaptive immune response with few naive Tcells versus T-cells, due to the involution of the thymus with age [16,17]. However, although elderly patients may exhibit fewer of the collected clinical symptoms in our study, their actual disease burden is very high, and overall mortality in the >80-year-old population has reached as high as 20.2% in Italy's pandemic data, and as low as 13% in South Korea [18,19]. Contrarily, while the <20-year-old groups in our study demonstrated low number of symptoms and disease burden, the population's actual fatality rate ranges between 0-0.2%, indicating a highly effective immune response to the virus [18,19]. We do note that symptoms in very young patients enrolled in this study may not be truly reflective of their actual experienced disease process. Similar studies have noted increased asymptomatic SARS-CoV-2 infection in younger patients and women, as compared to elderly patients and men [20,21]. Studies also noted a higher morbidity associated with elderly patients than young and middle-aged patients, and similarly determined the most common symptoms amongst all COVID-19 infected age groups as fever and cough with sputum [22,23]. Patients at this testing site received testing based on current or recent experience with influenza-like illness or clinical respiratory illness or having known COVID-19 patient contact. This could explain the findings of similar numbers of symptoms and disease burden in the Ab-negative study population, as they were likely experiencing some respiratory infection. Due to this, the data separating clinical features of COVID-19 positive and negative patients was difficult to distinguish. The Centers for Disease Control and Prevention have noted very similar clinical symptoms amongst COVID-19 and influenza-like illness [24]. This emphasizes the necessity to continue high testing output at clinical sites to track and attempt to manage the disease progression on a global scale to implement necessary social distancing and quarantine measures.

## Limitations:

Several assessed symptoms (shortness of breath, muscle/joint pain, fatigue) were not quantified for severity. Long-term patient outcomes such as mortality were not assessed. Type of testing method for each patient were not documented; the BIOSCIENCES and RayBiotech COVID-19 Ab tests have distinct test sensitivity and specificity which were not able to be accounted for in statistical analyses. The use of Ab tests to analyze COVID-19 positivity may present variation since the seroconversion time of these antibodies may take up to 15-days to develop [25,26].

## Conclusion

We report that COVID-19 disease burden and number of symptoms experienced were highest among the 40-69-year-old patients at the urgent care clinic in Volusia county, Florida. Those above the populations mean age of 46.4 years old were more likely to test positive for COVID-19.

## References

[1] https://www.who.int.

[2] Kamel Boulos MN, Geraghty EM (2020). Int J Health Geogr..

[3] www.worldometers.info/coronavirus/usa/florida/.

[4] Li R (2020). Science..

[5] Li C (2020). Emerg Infect Dis..

[6] Kimball A (2020). MMWR Morb Mortal Wkly Rep..

[7] Li JY (2020). Microbes Infect..

[8] Fu L (2020). J Infect..

[9] www.confirmbiosciences.com/coronavirus-test/.

[10] www.raybiotech.com/files/manual/CG-CoV-IgM-IgG.pdf..

[11] Bilcke J (2014). PLoS One..

[12] Jain VK (2013). N Engl J Med..

[13] https://datausa.io/profile/geo/volusia-county-fl..

[14] http://edr.state.fl.us/Content/populationdemographics/data/index.cfm..

[15] Simon AK (2015). Proc Biol Sci..

[16] Walker JM, Slifka MK (2010). Adv Exp Med Biol..

[17] Zinkernagel RM (2000). Phil. Trans. R. Soc. Lond. B Biol Sci.

[18] Onder G (2020). JAMA..

[19] https://cdn.onb.it/2020/03/COVID-19.pdf.pdf..

[20] Hu Z (2020). Sci China Life Sci..

[21] Yang R (2020). JAMA Netw Open..

[22] Liu K (2020). J Infect..

[23] Li LQ (2020). J Med Virol..

[24] https://www.cdc.gov/flu/symptoms/flu-vs-covid19.htm..

[25] Zhao J (2020). Clin Infect Dis..

[26] Deeks JJ (2020). Cochrane Database of Systematic Reviews.

